# Exploring the relationship between frequent cannabis use, belief updating under uncertainty and psychotic-like symptoms

**DOI:** 10.3389/fpsyt.2024.1309868

**Published:** 2024-07-24

**Authors:** Xinyi Liang, Maria-Mihaela Avram, Toni Gibbs-Dean, Edward Chesney, Dominic Oliver, Simiao Wang, Stiliyana Obreshkova, Tom Spencer, Amir Englund, Kelly Diederen

**Affiliations:** ^1^ Department of Psychosis Studies, Institute of Psychiatry, Psychology and Neuroscience, King’s College London, London, United Kingdom; ^2^ School of Medicine, Yale University, New Haven, CT, United States; ^3^ Department of Psychiatry, University of Oxford, Oxford, United Kingdom; ^4^ Department of Psychiatry, National Institute for Health and Care Research (NIHR) Oxford Health Biomedical Research Centre, Oxford, United Kingdom; ^5^ Addictions Department, Institute of Psychiatry, Psychology and Neuroscience, King’s College London, London, United Kingdom

**Keywords:** cannabis, psychosis, belief-updating, learning, online assessment

## Abstract

**Background:**

Cannabis users present an important group for investigating putative mechanisms underlying psychosis, as cannabis-use is associated with an increased risk of psychosis. Recent work suggests that alterations in belief-updating under uncertainty underlie psychosis. We therefore compared belief updating under uncertainty between cannabis and non-cannabis users.

**Methods:**

49 regular cannabis users and 52 controls completed the Space Game, via an online platform used for behavioral testing. In the task, participants were asked to predict the location of the stimulus based on previous information, under different uncertainty conditions. Mixed effects models were used to identify significant predictors of mean score, confidence, performance error and learning rate.

**Results:**

Both groups showed decreased confidence in high noise conditions, and increased belief updating in more volatile conditions, suggesting that they could infer the degree and sources of uncertainty. There were no significant effects of group on any of the performance indices. However, within the cannabis group, frequent users showed worse performance than less frequent users.

**Conclusion:**

Belief updating under uncertainty is not affected by cannabis use status but could be impaired in those who use cannabis more frequently. This finding could show a similarity between frequent cannabis use and psychosis risk, as predictors for abnormal belief-updating.

## 1 Introduction

Psychosis is characterized by symptoms such as hallucinations and delusions that can cause severe distress (1). Contemporary research on psychosis increasingly focuses on the investigation of non-clinical cohorts deemed to have heightened vulnerability to psychotic disorders (2) as well as those exhibiting subclinical phenomena reminiscent of psychotic symptoms (3). These groups are often more accessible than clinical groups and might reveal clinically relevant details about psychotic symptoms that can be observed in the absence of the confounding effects of factors such as medication (4). They may also enable us to identify markers that are predictive of psychosis-transition in risk groups (5).

A large body of research investigates people at clinical high risk for psychosis (CHR-P) who have an estimated overall transition rate to psychosis of 25% within a three-year timeframe (3). While this research has already revealed important insights, it is important to note that only a minority of people with a first episode of psychosis were previously managed/identified by early detection services aimed at CHR-P individuals (6, 7). This stresses the importance of investigating other groups of individuals who may be more vulnerable to, or present with risk factors for psychosis.

An established risk factor for psychosis is cannabis use (8). It is associated with an increased risk of psychotic disorders, particularly among adolescent users (8–12). Specifically, daily cannabis users were found to be three times more likely to experience a psychotic episode compared to non-users (13). One study showed that almost half of those presenting with cannabis-induced psychosis developed a psychotic disorder in the following three years (14), while another longitudinal study found a considerable association between cannabis use disorder and the subsequent development of psychotic unipolar depression and bipolar disorder (15). Finally, cannabis has been associated with alterations in dopamine release, the main neurotransmitter in psychosis, although results are mixed (16).

As such, cannabis users are a valuable group to investigate candidate mechanisms for psychosis. There is increasing evidence to suggest that psychotic symptoms result from key alterations in the way that individuals update their beliefs about uncertain outcomes (17–20). Belief-updating under uncertainty involves the adaptation to the constantly changing environment, by distinguishing relevant from irrelevant stimuli (21–23). In this regard, the term “expected uncertainty” (noise) refers to the predictable variability of the environment, while “unexpected uncertainty” refers to unpredictable changes (volatility) in the environment (23, 24). Successful adaptation requires a high rate of belief-updating under conditions of unexpected uncertainty, where new information could serve as evidence for an important change, while a low learning rate should be adopted under the conditions of expected uncertainty, where new information is an expected variation of the current belief (23). Studies that investigated belief-updating under uncertainty using probabilistic paradigms found an association with psychosis, particularly an overestimation of environmental volatility and failure to adapt to change in uncertainty (25, 26). These findings have also been replicated in cohorts with CHR-P (27–29).

While there is evidence supporting that the attribution of salience to insignificant events (referred to as aberrant salience), which may impact belief updating, is associated with cannabis use, research on belief updating in cannabis users (30) remains scarce. The only study to date that investigated belief-updating in cannabis-users, did not include high-volatility conditions, the processing of which is thought to be primarily affected in psychosis (31). More research is thus required to investigate how cannabis users adapt to noise and volatility and reveal whether there are any shared patterns with individuals on the psychosis spectrum. This study, therefore, aimed to investigate the association between cannabis use and belief-updating under conditions of noise and uncertainty in a general population sample. We used a novel task, designed to capture the key information needed to learn about uncertain outcomes (the mean, expected and unexpected uncertainty). A secondary aim was to explore if there would be a relationship between cannabis use, psychotic-like symptoms, and belief- updating under uncertainty.

## 2 Methods

All procedures in this study were approved by the King’s College London ethics committee (Cannabis users: LRU-20/21–20376, non-cannabis-users: MRA-19/20–19444) and all participants provided informed consent before taking part in the study. The collected data was processed in compliance with the United Kingdom General Data Protection Regulation (GDPR).

### 2.1 Participants

Participants were recruited online through the platform ‘Prolific’ (https://www.prolific.co), which is an online platform used for anonymous recruitment of research volunteers. The cannabis-using group (CU) was selected based on the completion of a pre-screening questionnaire on Gorilla (https://gorilla.sc), which assessed cannabis use status and medical history. The inclusion criteria were age 18–40, fluency in English, UK residence, and frequency of cannabis use of 3 times a week or more. The people who were currently undergoing treatment or taking medications for treating symptoms of anxiety and depression and those who had a current or past diagnosis of a mental health condition were excluded from the study. The control group was selected from a larger general population cohort through the same procedure as the cannabis users. The participants who reported never using cannabis (generally or continuously) and responded “not applicable” to the question about the type of cannabis used were selected as non- cannabis users (NCU). They were matched with the CU group by age, gender, ethnicity, device type and education.

### 2.2 Procedure

The volunteers who fulfilled the eligibility criteria were invited to participate in the study via Gorilla (gorilla.sc), an online platform that is used for behavioral experiments. The participants provided informed consent and completed a questionnaire on baseline demographics, before taking part in the study.

Both participant cohorts were part of a larger study, and data was collected on demographic information, mental health scales (for depression, anxiety, paranoia, psychometric schizotypy), and performance in a range of behavioral tasks, including the Space Game. After completing the study, the participants were reimbursed with £6.50 per hour spent and an additional payment of up to £2 for the score obtained in the Space Game.

### 2.3 Materials

#### 2.3.1 The Schizotypal Personality Questionnaire

This study included the scores on the Schizotypal Personality Questionnaire-Brief (SPQ) which is a 22-item self-reporting tool consisting of yes/no questions about 3 domains of schizotypal personality disorder: (i) Cognitive perceptual deficits (CPD), referring to belief-related traits such as magical thinking or paranoid ideation, (ii) Interpersonal Deficits, regarding social interactions, and (iii) Disorganization, which refers to unusual behavior or speech (32).

#### 2.3.2 The Peters Delusion Inventory

The Peters Delusion Inventory (PDI) is a 21-item measuring delusional ideation in the general population (33). The PDI is comprised of 11 components: persecution, suspiciousness, paranoid thinking, religiosity, grandiosity, paranormal beliefs, thought disturbances, negative self, depersonalization, catastrophic ideation and thought broadcast, ideation of reference and influence (33).

#### 2.3.3 The Space Game

The Space Game is a probabilistic learning task adapted from a previous study (34) designed as an interactive game in which participants are tested on their ability to adapt to changes in noise (expected uncertainty) and volatility (unexpected uncertainty) of the environment.

**Figure 1:**
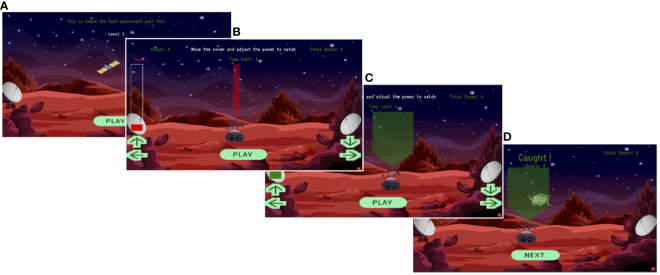
An overview of the Space Game. **(A)** Hint. The screen shows where the last piece of space junk fell. Please use this information to guess where the next piece will fall. **(B)** Move the rover. Move the rover to the place where you think the space junk will fall. On a computer use the keyboard arrows. On a smartphone, use the arrows on the touchscreen. **(C)** Adjust the power. Adjust the power, using the lowest setting that will allow you to catch the junk, using the up and down arrows. Once you have adjusted the power it will become green. **(D)** Feedback. The screen will show you where the junk landed, whether you caught it and if you caught it, how many points you gained.

In the task, the participants are instructed to collect space junk that falls from an unseen spaceship, by horizontally moving a rover (keys A,D or arrows left and right), which has an adjustable electromagnetic ‘beam’ (keys W,S or arrows up and down) that can collect the falling junk (**Figure 1**). By successfully collecting the falling space junk, participants can obtain points and the narrower the beam, the more points are added to the total score. The score is then converted to monetary rewards (section 2.2), which creates an incentive for participants to adapt to the uncertainty in the location of the space junk and thus obtain a higher score.

The task consists of 219 trials divided into 6 blocks (i.e. levels) (**Table 1**), which begin when the participant starts moving the rover and stop after 5 seconds have passed. At the beginning of each level, participants are given a cue of where the space junk fell previously so that they can adapt their prediction and move the rover to the predicted location of the next falling junk. This process is repeated in each trial. For a more precise prediction of the new location, participants can adjust the width of the rover beam (**Figure 1**). At the end of each trial, participants are given feedback about whether they were successful in catching the space junk, or whether they missed (**Figure 1**). The trial score is then calculated based on whether the space junk was collected and the precision of the beam width. Successful collection of the space junk indicates that the prediction of its location was correct, and a narrower beam width indicates a higher level of confidence in this prediction.

**Table 1:** Overview of the number of trials, uncertainty conditions and type of rewards in each level (block) of the Space Game.

	N trials	Mean of the distribution	SD of the distribution	Uncertainty condition	Reward Type
**Level 1** **(practice)**	3 (repeat until successful)	-0.07	0.045	N/A	NA
**Level 2**	10	0	0.12 or 0.03	No volatility--50% participants SD=0.12; 50% participants SD=0.03	Gains
**Level 3**	40	0	0.12, 0.03	No volatility; noise changed from high to low or vice versa	Gains
**Level 4**	40	0.35, -0.35	0.06	High volatility	Gains
**Level 5**	84	0.35, -0.35, 0.14, -0.21	0.12, 0.03	High volatility; noise switched between high and low	Gains
**Level 6**	42	0.35, -0.21	0.12, 0.03	High volatility; noise switched between high and low	Increased gains or losses

The noise and volatility of the falling junk are statistically manipulated by changing the standard deviation (SD) and mean, respectively, of the normal distribution by which the location of the falling junk is computed. These changes vary depending on the level of the game, as shown in **Table 1**. For example, in level 3, which simulates high noise conditions, the SD changed midway through the level, while the mean remained the same. Alternatively, at level 4, the SD was maintained throughout the level, while the mean changed midway through, simulating unexpected uncertainty (volatility). Level 5 combines high noise and high volatility, while level 6 adds the possibility to have higher gains with higher precision, but also losses when unsuccessful.

### 2.4 Outcome measures

For each participant, a set of performance metrics were computed based on performance in each trial. The most comprehensive performance metric which considers both participants’ learning of the mean and the noise is *score*. The score is zero when the space junk is not collected and ranges from one to ten depending on precision when the space junk is collected, with a smaller beam width corresponding to a higher score.

To investigate whether the participants learned to infer the main characteristics of the outcome distributions and task structure that had to be learned, the following outcome measures were used. First, to successfully complete the task, the participants had to keep track of the mean of the normal distribution from which the space junk location was computed. As the most accurate prediction would be the mean of this distribution, *Performance error (PerfE)* was calculated as the absolute difference between the participant’s location in a trial and the true mean of the distribution. Secondly, as participants should keep track of the noise of the underlying distribution, using a large beam width when the SD of the underlying normal distribution was higher, *Confidence* was quantified as the inverse of beam width, with a greater beam width indicating a lower level of confidence, and thus a higher subjective uncertainty of the exact location of the falling space junk. To determine if participants were able to infer the relative noise level, confidence was compared for trials with low vs high SD (based on the underlying normal distribution from which the space junk was drawn), thus reflecting participants’ ability to adapt to the noise. Finally, to determine if participants could infer changes (i.e., volatility) in the underlying normative distributions, and whether they could ‘reset’ their beliefs about the junk locations when such a change occurred, *Learning rates (LR)* were calculated by dividing the change in rover position by the difference between the participant position and the location of the falling space junk (i.e., the prediction error), as it was done in a similar task previously (34). The trial-wise LRs were adjusted to deal with that were outlier values (over 1) and negative values that occurred due to a change in the direction opposite to the location of the falling space junk. The values that were lower than 0 were recoded to 0, while the values higher than 1 were recoded to 1. From the adjusted LR values, a new categorical variable was created, separating trial-wise learning rates in 3 different categories based on how much beliefs were updated in each trial. The non-updates occurred when LR was between 0 and 0.1 (inclusive), moderate belief updates were attributed to trials in which LRs were between 0.1 and 0.9, and total updates were attributed to LRs equal to or higher than 0.9, as done by Nassar et al. (35).

### 2.5 Data cleaning and analysis

The experiments included two attention checks, in which participants were asked to select a particular option. The attention checks had the purpose of identifying participants that did not interact sufficiently with the study. In the data cleaning process, the participants who failed the attention checks, did not complete the task, or completed it repeatedly were excluded from the study. Additionally, participants that were believed to “spam through” the space task were excluded from the analysis, based on the following criteria: (i) no movement of the rover in 10% or more of all trials and (ii) completion of 10% or more trials in under 1000ms.

All data was analyzed using R version 4.2.3. Frequency tables were extracted using the *tidyverse* and *janitor* libraries. Between-group comparisons were done for demographic data, including matching variables. T-tests and Mann-Whitney U-tests were used for numerical parametric and non-parametric data, respectively. Chi-square tests were used for comparing categorical variables.

Linear mixed effects (LME) models (*lme4* library in R) were used to analyze the effects of group and level on the performance metrics, as well as the effects of cannabis frequency, type, and last use on mean scores at different levels. LME models were chosen over multiple or multivariate linear regression models, due to the possibility to add a random effect that adjusted for the repeated measures. For each performance metric, a model was selected from a range of mixed models, starting from the least complicated model that included one fixed effect (group) and two random effects (participant and counterbalancing condition nested within level) to account for multiple measures and varying versions of the game. More complex models included a larger number of fixed effects and/or interaction effects. The most adequate models were selected based on the Akaike information criterion (AIC), which is a measure indicating how well a model fits the data (36), with lower AIC values indicating a lower amount of information loss. The models including different combinations of fixed effects were compared using a X^2^-test, and the models that were significantly better than others were selected for analysis. When a X^2^-test was not possible or there were no significant differences between similar models, the model with a numerically lower AIC value was chosen (37).

To analyze which variables predicted mean score and PerfE in different uncertainty conditions, the values were averaged by level per participant using the *tidyverse* library, and the final analysis included contrasts between levels 3–4, 4–5, and 5–6, as measures of adaptation to changes in uncertainty. To analyze confidence, the participant-averaged data was grouped by the type of SD value - new categorical variable - in which a TRUE value was attributed to SD=0.12, while a FALSE value was attributed to SD = 0.06 or 0.03. For the belief-update type analysis, a multinomial logistic regression with random effects was used (*mclogit* library), in which the trial-wise update type was an outcome variable, and the best model fit was chosen as described previously.

Data from levels 1 and 2 were not included in the final analysis, due to level 1 being a practice level and level 2 having had distributions that differed across participants (to average out potential priming effects of starting with a distribution with a low vs high noise level; **Table 1**). Since level 6 included the possibility of obtaining a higher score, but also losing points, the score range differed from other levels and thus scaling was applied, using the *scales* library in R. Statistical significance was set for alpha = 0.05, and the Wald method (38) was used to estimate degrees of freedom in LME and multinomial logistic models.

## 3 Results

### 3.1 Participant characteristics

A total of 79 cannabis users and 539 individuals from the general population completed the study and attempted the Space Game. As a result of the data-cleaning process, 30 (37.9%) cannabis users and 106 (19.7%) participants from the general population were excluded. After applying the filter for cannabis use and excluding the participants that did not have matching demographics with CU (device type, ethnicity) the control group consisted of 53 NCU. During the analysis, another participant was excluded from the control group due to apparent participation in the cannabis study. As a result, 49 CU (mean age (SD) = 28.4(6.36) years, male = 63.3%) and 52 NCU (mean age (SD) =26.8(5.9) years, male =51.9%) were included in the analysis. There were no significant differences in age, gender, ethnicity, education level and device type between the final included two groups (**Table 2**). The majority (98%) of the participants completed the SPQ, the score of which did not differ significantly between the groups (Mann Whitney U =1460.5, p = 0.099). There was a significant difference in the score on the CPD sub-scale of the SPQ between the groups, with cannabis users obtaining a significantly higher score than the NCU controls (Mann Whitney U = 1669.5, p = 0.001). Cannabis users scored significantly higher on the PDI scale (Mann Whitney U = 1546.5, p = 0.040). From the PDI subscales, cannabis users had a significantly higher conviction score (Mann Whitney U = 1543.5, p = 0.043), and had a tendency for a higher preoccupation score, which did not reach statistical significance (Mann Whitney U = 1532, p = 0.051).

**Table 2:** Participant demographics.

Participant characteristics	Cannabis users	Non-cannabis users	P-value
Age, years			
Mean (SD)	28.4 (6.36)	26.8 (5.9)	0.18
Gender, n (%)			
Male	31 (63.3)	27 (51.9)	0.39
Female	18 (36.7)	25 (48.1)	
Ethnicity,n (%)			
Asian	7 (14.3)	8 (15.4)	0.71
Black	5 (10.2)	3 (5.8)	
White	37 (75.5)	41 (78.8)	
Education,n (%)			
Highschool (on-going)	0 (0)	1 (1.9)	0.2
Highschool (finished)	8 (16.3)	15 (28.8)	
Professionaltraining (on-going)	3 (6.1)	0 (0)	
Professionaltraining (finished)	2 (4.1)	4 (7.7)	
University (on-going)	8 (16.3)	8 (15.4)	
University (finished)	15 (30.6)	16 (30.8)	
Post-graduateuniversity (on-going)	3 (6.1)	0 (0)	
Post-graduateuniversity (finished)	10 (20.4)	8 (15.4)	
Devicetype,n (%)			
Computer	44 (89.8)	44 (84.6)	0.63
Mobile	5 (10.2)	8 (15.4)	
SPQscore			
Mean (SD)	8.78 (4.20)	7.44 (5.46)	0.099
Cognitiveperceptualdeficitsscore	2.55 (1.97)	1.4 (1.71)	0.001**
Interpersonaldeficitsscore	4.24 (2.33)	4.16 (2.68)	0.94
Disorganisationscore	1.98 (1.73)	1.88 (2.03)	0.48
PDIscore			
Mean (SD)	45.14 (36.2)	32.33 (31.3)	0.040*
Convictionscore	15.57 (12.67)	10.49 (9.84)	0.043*
Distressscore	11.71 (9.93)	9.29 (9.62)	0.145
Preoccupationscore	13.33 (11.77)	9.18 (9.63)	0.051

### 3.2 Space task performance in cannabis-users vs non-cannabis users

#### 3.2.1 Overall performance (score)

**Figure 2:**
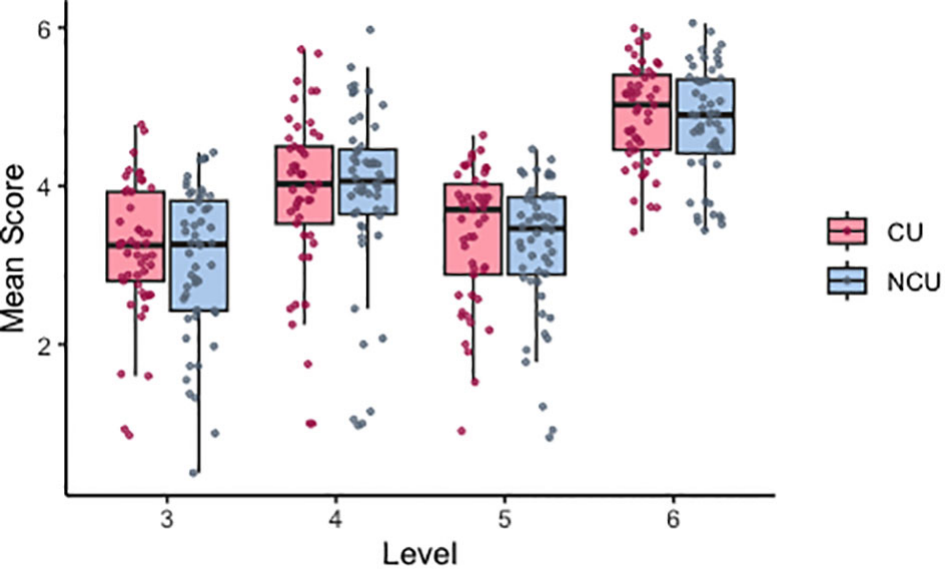
Distribution of mean scores in all participants across levels 3–6. Individual datapoints indicate values averaged by participant. Level 3 = high noise/low volatility, level 4 = low noise/high volatility, level 5 = high noise/high volatility, level 6 = high noise/high volatility inc. negative rewards. CU, cannabis users; NCU, non- cannabis users.

The best-fitted model for mean score included participant and counterbalancing condition nested within level as random effects and group, level, total SPQ score, and total PDI score as fixed effects. A model including an interaction term did not significantly improve the model fit (X2 = 0.372, p = 0.542). There was no significant effect of the group, SPQ total score or PDI total score on predicting mean scores (**Figure 2**). Contrast analyses showed significant differences across mean scores between levels (level 4 vs level 3: Beta (SE) = 0.67(0.05), p<0.001; level 5 vs level 4: Beta (SE) = 0.56(0.06), p<0.001; level 6 vs level 5: Beta (SE) = 1.05(0.05), p<0.001). These results indicate that advancing levels (increasing levels of uncertainty and negative rewards) predicted changes in scores when adjusting for group, SPQ score, and PDI score.

#### 3.2.2 Performance error

The best-performing model for PerfE included participant and counterbalancing condition nested within level as random effects and level, PDI total score and an interaction between group and SPQ total score as fixed effects. A model including an interaction term significantly improved the model fit (X2 = 4.42, p = 0.036). Contrast analysis showed significant differences in PerfE between levels (level 4 vs. level 3: Beta (SE) = -0.72 (0.13), p <0.001; level 5 vs. level 4: Beta (SE) = 0.65 (0.15), p < 0.001; level 6 vs. level 5: Beta (SE) = 1.81 (0.13), p < 0.001). These results show that participants had high performance errors (i.e., low accuracy) in the initial high noise level (level 3) when they were still learning about high noise distributions in the task and where even the best predictions will have relatively high-performance errors due to the imposed noise.

**Figure 3:**
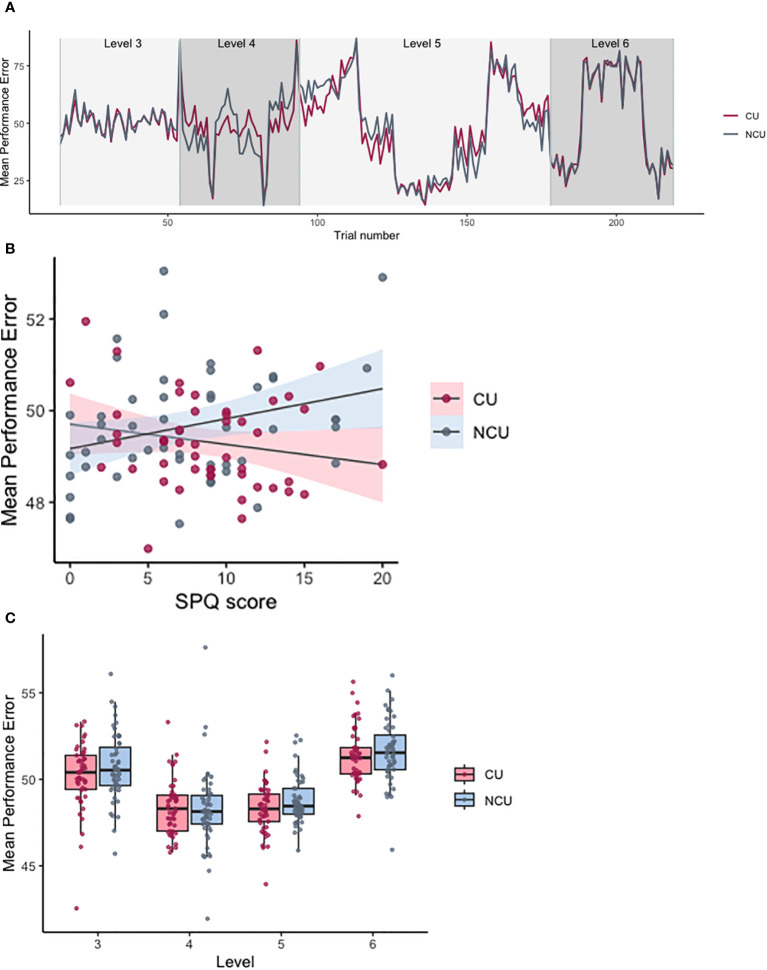
Performance errors for levels 3–6 of the Space Game. **(A)** Mean performance errors across levels. Individual datapoints indicate values averaged by participant. Level 3 = high noise/low volatility, level 4 = low noise/high volatility, level 5 = high noise/high volatility, level 6 = high noise/high volatility inc. negative rewards. **(B)** Scatterplot indicating the effect of the group-by-SPQ interaction on PerfE. Shaded areas stand for standard errors. **(C)** Trial-wise performance errors averaged by trial by group, starting from trial 14 (level 3) till trial 219 (end). CU, cannabis users; NCU, non-cannabis users.

**Figure 4:**
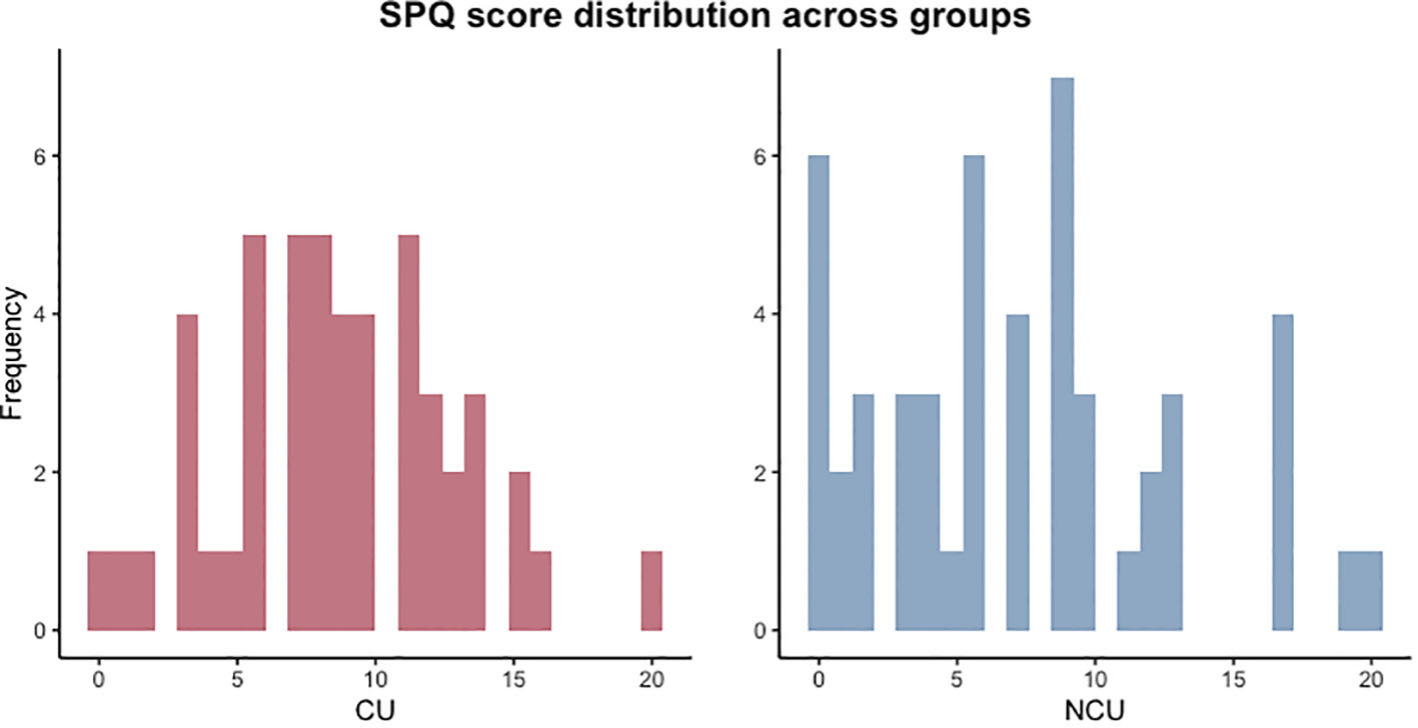
SPQ total score histograms for cannabis and non-cannabis users.

Performance errors decreased in the next level when the noise reduced but some volatility was imposed (level 4), and then started increasing again in subsequent conditions (level 5) that had higher noise and more frequent mean changes (volatility) and when negative rewards were set (level 6) (**Figures 3A, C**) thus making it harder to keep track of the mean of the underlying distributions. The interaction between group and SPQ significantly predicted PerfE (Beta (SE) = 0.10 (0.05), p = 0.034, **Figure 3B**). *Post-hoc* t-tests for SPQ total score indicated that there was a significant difference between the two groups (t(df) = 2.75 (373.15), p = 0.006), where CU displayed higher mean SPQ total score (mean = 8.78) than NCU (mean = 7.44). To further understand the significant interaction, histograms for SPQ total scores were also plotted for CU and NCU (**Figure 4**.) *Post-hoc* correlation analyses revealed a non-significant correlation between average PerfE and SPQ scores in NCU (Kendall’s tau = 0.173, p = 0.085), and a non-significant correlation between PerfE and SPQ scores in CU (Kendall’s tau = - 0.145, p = 0.151).

#### 3.2.3 Confidence

**Figure 5:**
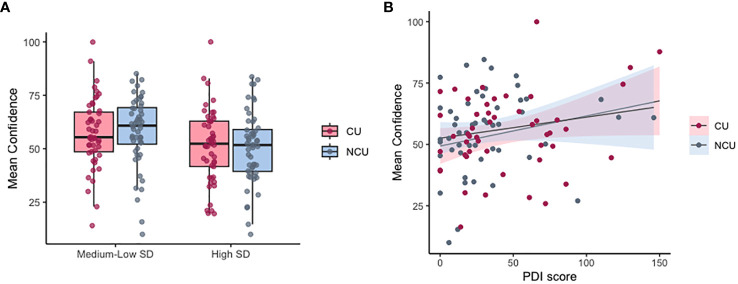
Mean confidence in cannabis and non-cannabis users. **(A)** Mean confidence by group in high noise conditions (SD=0.12) vs medium-low noise (SD = 0.03/0.06). **(B)** Relationship between confidence and PDI score across groups (CU, cannabis users; NCU, non-cannabis users). Individual data points are values averaged by participant per SD condition.

The model predicting confidence that showed the best fit for the data included group, SD as a categorical variable (high SD/low SD), SPQ total score, and PDI total score as fixed effects and participant and counterbalancing condition nested within level as random effects. There was no significant effect of group and SPQ score on confidence (Beta (SE)NCU = 1.26(3.19), p=0.69; Beta (SE)SPQ = -0.61(0.41), p=0.136). However, there were significant effects of SD category, showing that the high SD category predicted significantly lower confidence (Beta (SE) = -6.73(0.77), p<0.001). This indicates that participants had significantly lower confidence in their decision-making in high noise conditions, compared to low noise conditions (**Figure 5A**). Additionally, higher PDI scores significantly predicted higher average confidence regardless of noise condition or cannabis use (Beta (SE) = 0.16(0.06), p = 0.007, **Figure 5B**).

#### 3.2.4 Learning rate

The best-fitted multinomial model for predicting the type of updates (non, moderate, full) included group, level, and gender as fixed effects and participant and counterbalancing condition nested within level as random effects. None of the predictors had a significant effect on the difference between non-updates and moderate updates. Gender predicted a significantly lower difference between total updates and moderate updates in males (OR(CI) = 0.54(0.43 – 0.68), p < 0.001). Contrast analyses also revealed a significant effect of level on the difference between total updates and moderate updates (level 4 vs level 3: OR (CI) = 4.87(2.95–8.04), p < 0.001; level 5 vs level 4: OR(CI) = 1.78 (1.22–2.59), p = 0.003). These findings indicate that (i) males had a lower frequency of total updates relative to moderate updates, than women and (ii) participants had a higher frequency of total updates relative to moderate updates when advancing between levels 3 and 4, and between levels 4 and 5, indicating that they adapted their learning rate to increased volatility, when previous information was no longer informative.

### 3.3 Task performance in cannabis user subgroups

#### 3.3.1 Mean score

**Figure 6:**
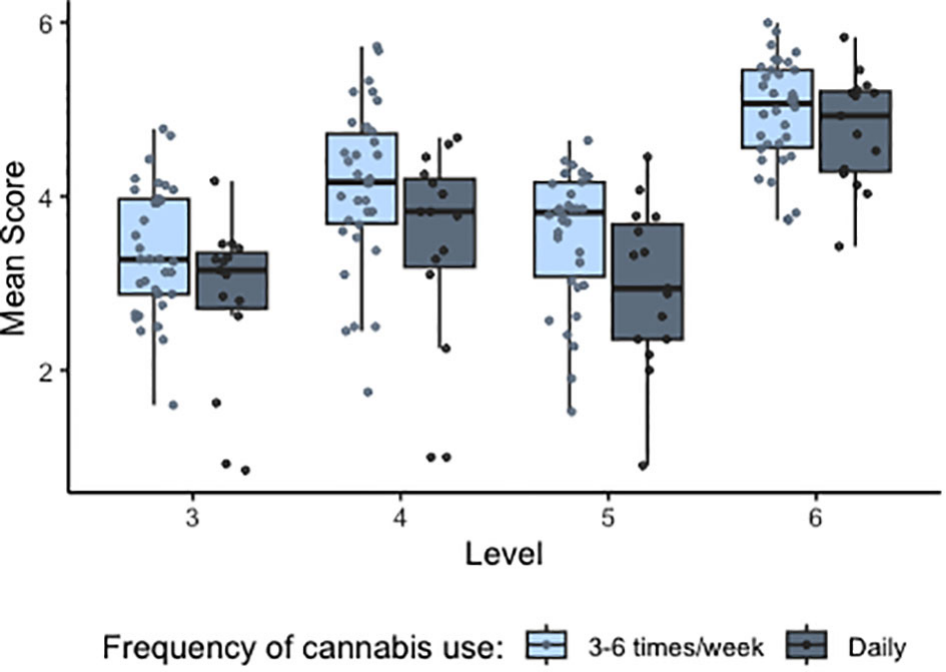
Distribution of mean scores in cannabis users by frequency of cannabis use, across levels 3–6. Individual datapoints indicate values averaged by participant.

The best-fitted LME model for predicting mean score included participant and counterbalancing condition nested within level as random effects and cannabis type (strong cannabis/hash/resin/weak cannabis), frequency of cannabis use (daily/three times a week or more often), last use (under 24 hours/over 24 hours), level, and gender as fixed effects. The model was not significantly improved by adding any interaction effects (X^2^ = 2.004, p=0.57). There were no significant effects of cannabis type or last use on score. However, cannabis use frequency significantly predicted mean scores, with a higher frequency (daily) predicting a significantly lower mean score than using cannabis between 3–6 times a week (Beta (SE) = -0.39 (0.196), p = 0.047, **Figure 6**). There was a significant effect of gender on performance and a higher score was predicted for males than for females (Beta (SE) = 0.40(0.18), p = 0.029).

#### 3.3.2 Performance error

The best-fitted model for predicting PerfE included participant and counterbalancing condition nested within level as random effects and frequency, level, last use, and cannabis type as fixed effects. Contrast analyses demonstrated a significant effect on PerfE between levels (level 4 vs. level 3: Beta (SE) = -0.617 (0.187), p < 0.001; level 5 vs. level 4: Beta (SE) = 0.598 (0.216), p = 0.006; level 6 vs. level 5: Beta (SE) = 1.866 (0.187), p < 0.001). There was no significant effect of cannabis frequency, cannabis type and last use on PerfE.

#### 3.3.3 Confidence

The best-fitted LME model for predicting confidence included participant and counterbalancing condition nested within level as random effects and cannabis type, frequency of cannabis use, SD category, last use, and PDI total score as fixed effects. There was no significant effect on frequency, last use, and cannabis type, while the significant effects of SD category and PDI scores were preserved, with high SD predicting significantly lower confidence (Beta (SE) = -6.48 (0.99), p < 0.001) and PDI total score predicting significantly higher confidence (Beta (SE) = 0.124 (0.059), p = 0.038).

#### 3.3.4 Learning rate

The best-fitted multinomial mixed effects model for belief update categories had gender, level, frequency, cannabis type, and last use as fixed effects and participant and counterbalancing condition nested within level as random effects. None of the fixed effects significantly predicted the difference between non-updates and moderate updates. There was a significant effect of gender, as shown in the main analysis, indicating that male gender predicted a significantly smaller number of total updates relative to moderate updates, as opposed to female gender (OR(CI) = 0.458 (0.327–0.642), P<0.001). Between-level contrast predictors showed significant effects on the difference between total updates and moderate updates, similar to the main analysis (level 4 vs level 3: OR(CI) = 6.28(2.65–14.87), p < 0.001; level 5 vs level 4: OR (CI) = 2.05(1.091–3.87), p = 0.026.

**Figure 7:**
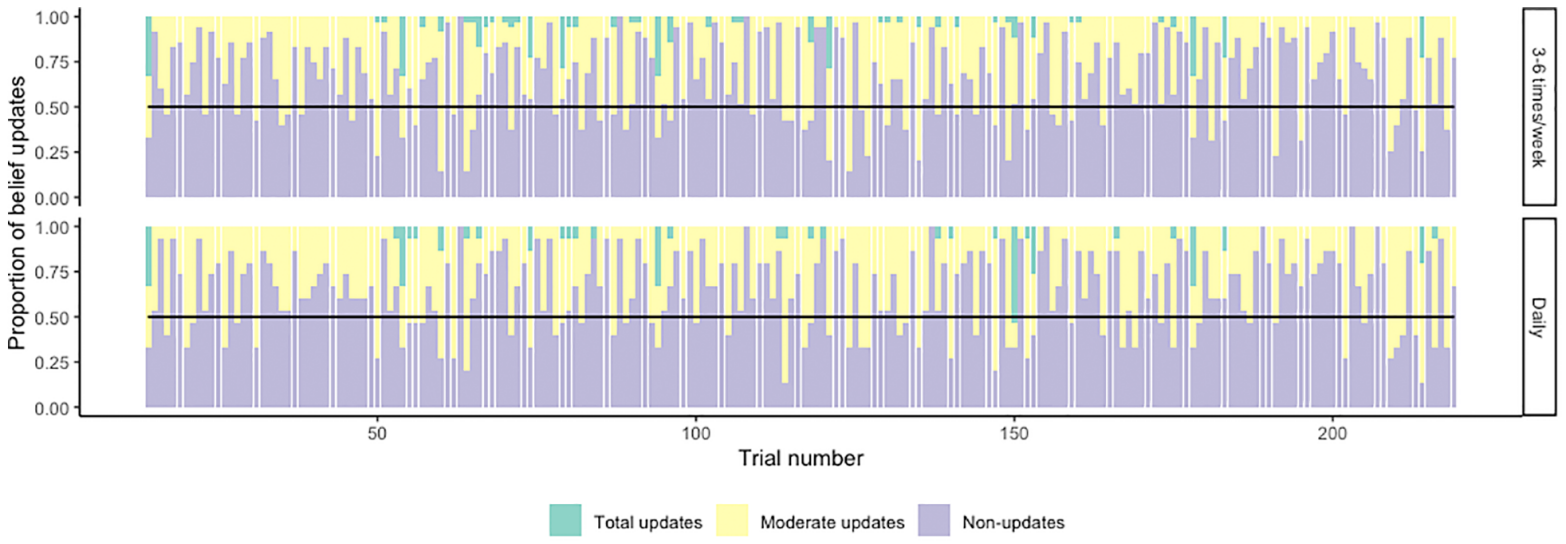
Belief update types in cannabis users. Bars indicate proportions of total updates **–** moderate updates – non updates averaged by trial, starting from trial 14 (level 3) and ending in trial 219 (end of level 6). The top plot indicates belief updates in participants who used cannabis 3–6 times per week, while the bottom plot shows belief updates in participants who used cannabis daily.

Cannabis frequency had a trend-level effect on predicting belief updates. That is, daily cannabis use predicted a smaller number of total updates relative to moderate updates (OR(CI) = 0.68(0.46–1.01), p = 0.059, **Figure 7**), when compared to a less frequent use (3- 6 days a week). This indicates that people who used cannabis more frequently had a tendency to completely update their beliefs less than those who used cannabis less frequently.

## 4 Discussion

### 4.1 Task performance and learning

This study aimed to investigate the relationship between cannabis use, belief-updating and psychotic-like experiences using a novel online behavioral task. The task was designed to manipulate noise and volatility conditions independently and combined to simulate real-life belief-updating. Results indicate that performance (score), performance error, confidence and belief updates differed under changing uncertainty conditions, with a pattern of an increase in learning rates under conditions of unexpected uncertainty, which is in line with previous findings in probabilistic learning tasks (29, 31, 39–41). Although differences were present across uncertainty conditions, none of the performance metrics differed between CU and NCU.

Previous studies using the Iowa Gambling task (IGT) found decreased performance in CU (30) or late-onset cannabis use compared to early onset (42) which was associated with poorer decision-making. Additionally, chronic cannabis users were found to be less sensitive to losses compared to healthy controls assessed with IGT (43), an effect that was also observed in schizophrenia patients in a study investigating IGT performance (44), which suggests that there might be shared underlying behavioral patterns between cannabis use and psychosis. However, the present study showed no significant difference between the NCU and CU, or an effect of cannabis frequency, type, or recency in performance the win/loss condition of the task (level 6), which employed a risk assessment-based decision-making factor (choosing higher and risky, over lower and certain rewards).

One of the secondary aims of this study was to explore the effects of PDI and SPQ scores on task performance in the two groups. The results showed that performance error was predicted by SPQ score, depending on cannabis use, in particular, cannabis use attenuated performance error in individuals scoring high on the SPQ, as opposed to non-cannabis use. Relevant to this finding, a previous study found that aberrant salience mediated the relationship between cannabis use and SPQ scores (45). Aberrant salience refers to abnormal attribution of salience, significance, or value to otherwise neutral stimuli (46) which might occur before full-blown psychosis (47, 48). Our results uncovered a new layer in the relationship between schizotypy, cannabis use, and belief updating, in particular that cannabis use could have opposing effects on belief-updating in those at the higher end of the schizotypy spectrum. Interestingly, a previous study found improvements in cognition associated with cannabis use prior to the first psychotic episode, as opposed to deteriorating effects on healthy controls (49). This raises the question of whether cannabis use interacts differently with cognition depending on psychotic-like experiences, which is in line with our finding. Alternatively, this finding could be explained by the significant difference in SPQ scores between cannabis users and non-cannabis users, with cannabis users displaying significantly higher SPQ scores, and lower variation in scores, than non-cannabis users. The pattern for SPQ histograms for these two groups were not similar, with NCU showing more extreme values towards the lower ends of SPQ scores than CU, which may have driven the significant interaction between SPQ score and performance error for the NCU compared to the CU. To further understand the results, Bayesian modelling methods could be used to estimate perceived environmental volatility (25) which could offer a better insight into this finding.

Another exploratory aim of the study was to evaluate the effects of cannabis type, frequency, and last use on belief-updating under uncertainty. The results showed that individuals who used cannabis more frequently performed poorer than those who used cannabis less frequently. These differences did not seem related to the cannabis-users’ ability to learn the mean or the noise of the distributions from which the space junk was drawn, as performance error and confidence were not predicted by cannabis frequency. However, we found a tendency of cannabis users who had a higher frequency of cannabis consumption to use fewer total updates in learning. Since the type of update is indicative of the participants’ ability to detect changes in the environment, these findings could be interpreted as tendency towards altered adaptation to environmental volatility by those who used cannabis more frequently, which is a behavioral pattern similar to those seen in individuals with psychotic symptoms (19, 20, 50). Nevertheless, the evidence in this study is not sufficient to infer this overlap and warrants further investigation into the relationship between heavy cannabis use, psychotic symptoms, and adaptation to volatility.

### 4.2 Limitations

Despite online recruitment being a great strength in behavioral studies, enabling accessibility to a larger population, and thus a greater generalizability of the results, the limitation of using this approach is that participants cannot be monitored for false reports or inaccurate interaction with the task. While a strength of our study was the data cleaning process, which removed a significant minority of users who did not complete the task with sufficient care, this also led to data loss which could have had an influence on the final results. Another limitation is that the study did not consider the possible effects of age of onset and amount of cannabis use, which could have offered greater insights into the cannabis subgroup analysis, since both age of onset and amount of cannabis use have been associated with increased psychosis risk (9, 51, 52). It is also worth mentioning that self-reported cannabis use is not always accurate, and the correlation with objective measures is limited in some populations (53), especially since the concentration of Δ9-tetrahydrocannabinol in different types of cannabis products can be substantially different (54). Furthermore, our analysis did not adjust for nicotine use, which was previously associated with impaired uncertainty processing in reinforcement learning (55) and could have had confounding effects on the cannabis frequency results since cannabis is often co-administered with tobacco (56). Additionally, the employment of any mental health disorder as an exclusion criterion might result in a more robust cannabis group as they reported frequent exposure to cannabis while remaining illness-free. The primary intent of employing this criterion was directed towards disentangling the effects of cannabis from mental health disorders. The focus was specifically on examining the impact of cannabis on belief updating under different uncertainty conditions. However, given the robust evidence indicating a strong positive correlation between the frequency of cannabis use and the likelihood of experiencing mental health disorders (13, 57), this exclusion criterion can be problematic. It is also essential to acknowledge that the present sample may not be entirely representative due to this selection criterion. Furthermore, it is worth noting that the Space Game is a gamified task, thus participants’ game use/habits might be a reasonably important factor that is nevertheless lacking in the present study. Thus, future work should collect data on participants’ game use experience and implement it into the matching procedures accordingly. Finally, although the novel Space Game used in the present study lends itself very well to capturing outcome measures without employing complex computational modelling, the Hierarchical Gaussian Filtering (HGF)—Jumping Gaussian Estimation Task can be useful in gaining deeper understanding of the impact of both volatility and noise on belief updating. This could be addressed in further studies.

To summarize, although we found no significant effect of cannabis use overall, there was a significant effect of cannabis frequency on task performance with more frequent users performing worse. These findings warrant further research investigating the relationship between frequency of cannabis use, psychosis risk, and aberrant belief- updating. It is also imperative to interpret our findings within the context of their exploratory nature. While the results offer insights into the relationship between cannabis use, belief-updating and psychotic-like experiences, it is important to recognize that these findings are hypothesis-generating rather than conclusive.

## Data availability statement

The raw data supporting the conclusions of this article will be made available by the authors, without undue reservation.

## Ethics statement

The studies involving humans were approved by Research Ethics Committee Kings College London. The studies were conducted in accordance with the local legislation and institutional requirements. The participants provided their written informed consent to participate in this study.

## Author contributions

XL: Formal analysis, Visualization, Writing – original draft, Writing – review & editing. MA: Formal analysis, Visualization, Writing – original draft, Writing – review & editing. TG: Conceptualization, Data curation, Formal analysis, Investigation, Methodology, Software, Supervision, Writing – original draft, Writing – review & editing. EC: Methodology, Supervision, Writing – review & editing. DO: Methodology, Supervision, Writing – review & editing. SW: Conceptualization, Data curation, Investigation, Writing – original draft. SO: Conceptualization, Data curation, Investigation, Writing – original draft. TS: Conceptualization, Funding acquisition, Methodology, Resources, Supervision, Writing – review & editing. AE: Conceptualization, Funding acquisition, Investigation, Methodology, Resources, Supervision, Writing – review & editing. KD: Conceptualization, Formal analysis, Funding acquisition, Investigation, Methodology, Project administration, Resources, Software, Supervision, Writing – review & editing.

## References

[1] American Psychiatric Association D, Association AP. Diagnostic and statistical manual of mental disorders: DSM-5 Vol. 5. Washington, DC: American psychiatric association (2013). doi: 10.1176/appi.books.9780890425596

[2] Seidman LJ Nordentoft M. New targets for prevention of schizophrenia: is it time for interventions in the premorbid phase? Schizophr Bull. (2015) 41:795–800. doi: 10.1093/schbul/sbv050 25925393 PMC4466192

[3] De Pablo GS Radua J Pereira J Bonoldi I Arienti V Besana F. Probability of transition to psychosis in individuals at clinical high risk: an updated meta-analysis. JAMA Psychiatry. (2021) 78:970–8. doi: 10.1001/jamapsychiatry.2021.0830 PMC8281006 34259821

[4] Van Haren NE Cahn W Pol HEH Kahn RS. Confounders of excessive brain volume loss in schizophrenia. Neurosci Biobehav Rev. (2013) 37:2418–23. doi: 10.1016/j.neubiorev.2012.09.006 23000300

[5] Koutsouleris N Riecher-Rössler A Meisenzahl EM Smieskova R Studerus E Kambeitz-Ilankovic L. Detecting the psychosis prodrome across high-risk populations using neuroanatomical biomarkers. Schizophr Bull. (2015) 41:471–82. doi: 10.1093/schbul/sbu078 PMC4332937 24914177

[6] Ajnakina O Morgan C Gayer-Anderson C Oduola S Bourque F Bramley S. Only a small proportion of patients with first episode psychosis come via prodromal services: a retrospective survey of a large UK mental health programme. BMC Psychiatry. (2017) 17:1–9. doi: 10.1186/s12888-017-1468-y 28841826 PMC5574213

[7] McGorry PD Hartmann JA Spooner R Nelson B. Beyond the “at risk mental state” concept: transitioning to transdiagnostic psychiatry. World Psychiatry. (2018) 17:133–42. doi: 10.1002/wps.20514 PMC5980504 29856558

[8] Hasan A von Keller R Friemel CM Hall W Schneider M Koethe D. Cannabis use and psychosis: a review of reviews. Eur Arch Psychiatry Clin Neurosci. (2020) 270:403–12. doi: 10.1007/s00406-019-01068-z 31563981

[9] Marconi A Di Forti M Lewis CM Murray RM Vassos E. Meta-analysis of the association between the level of cannabis use and risk of psychosis. Schizophr Bull. (2016) 42:1262–9. doi: 10.1093/schbul/sbw003 PMC4988731 26884547

[10] Robinson T Ali MU Easterbrook B Hall W Jutras-Aswad D Fischer B. Risk-thresholds for the association between frequency of cannabis use and the development of psychosis: a systematic review and meta-analysis. Psychol Med. (2022) 53:3858. doi: 10.1017/S0033291722000502 PMC10317818 35321777

[11] Kiburi SK Molebatsi K Ntlantsana V Lynskey MT. Cannabis use in adolescence and risk of psychosis: Are there factors that moderate this relationship? A systematic review and meta-analysis. Subst Abus. (2021) 42:527–42. doi: 10.1080/08897077.2021.1876200 33617756

[12] Oliver D Chesney E Cullen AE Davies C Englund A Gifford G. Exploring causal mechanisms of psychosis risk. Neurosci Biobehav Rev. (2024) 162:105699. doi: 10.1016/j.neubiorev.2024.105699 38710421 PMC11250118

[13] Di Forti M Quattrone D Freeman TP Tripoli G Gayer-Anderson C Quigley H. The contribution of cannabis use to variation in the incidence of psychotic disorder across Europe (EU-GEI): a multicentre case-control study. Lancet Psychiatry. (2019) 6:427–36. doi: 10.1016/S2215-0366(19)30048-3 PMC7646282 30902669

[14] Arendt M Rosenberg R Foldager L Perto G Munk-Jørgensen P. Cannabis-induced psychosis and subsequent schizophrenia-spectrum disorders: follow-up study of 535 incident cases. Br J Psychiatry. (2005) 187:510–5. doi: 10.1192/bjp.187.6.510 16319402

[15] Jefsen OH Erlangsen A Nordentoft M Hjorthøj C. Cannabis use disorder and subsequent risk of psychotic and nonpsychotic unipolar depression and bipolar disorder. JAMA Psychiatry. (2023) 80:803–10. doi: 10.1001/jamapsychiatry.2023.1256 PMC10209828 37223912

[16] Bloomfield MAP Hindocha C Green SF Wall MB Lees R Petrilli K. The neuropsychopharmacology of cannabis: A review of human imaging studies. Pharmacol Ther. (2019) 195:132–61. doi: 10.1016/j.pharmthera.2018.10.006 PMC6416743 30347211

[17] Fromm SP Wieland L Klettke A Nassar MR Katthagen T Markett S. Computational mechanisms of belief updating in relation to psychotic-like experiences. Front Psychiatry. (2023) 14:1170168. doi: 10.3389/fpsyt.2023.1170168 37215663 PMC10196365

[18] Sheffield JM Suthaharan P Leptourgos P Corlett PR. Belief updating and paranoia in individuals with schizophrenia. Biol Psychiatry Cognit Neurosci Neuroimaging. (2022) 7:1149–57. doi: 10.1016/j.bpsc.2022.03.013 PMC9827723 35430406

[19] Reed EJ Uddenberg S Suthaharan P Mathys CD Taylor JR Groman SM. Paranoia as a deficit in non-social belief updating. Elife. (2020) 9:e56345. doi: 10.7554/eLife.56345 32452769 PMC7326495

[20] Fromm S Katthagen T Deserno L Heinz A Kaminski J Schlagenhauf F. Belief updating in subclinical and clinical delusions. Schizophr Bull Open. (2023) 4:sgac074. doi: 10.1093/schizbullopen/sgac074 PMC11207849 39145350

[21] Adams RA Stephan KE Brown HR Frith CD Friston KJ. The computational anatomy of psychosis. Front Psychiatry. (2013) 4:47. doi: 10.3389/fpsyt.2013.00047 23750138 PMC3667557

[22] Behrens TEJ Woolrich MW Walton ME Rushworth MFS. Learning the value of information in an uncertain world. Nat Neurosci. (2007) 10:1214–21. doi: 10.1038/nn1954 17676057

[23] Soltani A Izquierdo A. Adaptive learning under expected and unexpected uncertainty. Nat Rev Neurosci. (2019) 20:635–44. doi: 10.1038/s41583-019-0180-y PMC6752962 31147631

[24] Bland AR Schaefer A. Different varieties of uncertainty in human decision-making. Front Neurosci. (2012) 6:85. doi: 10.3389/fnins.2012.00085 22701401 PMC3370661

[25] Katthagen T Fromm S Wieland L Schlagenhauf F. Models of dynamic belief updating in psychosis—a review across different computational approaches. Front Psychiatry. (2022) 13:814111. doi: 10.3389/fpsyt.2022.814111 35492702 PMC9039658

[26] Gibbs-Dean T Katthagen T Tsenkova I Ali R Liang X Spencer T. Belief updating in psychosis, depression and anxiety disorders: A systematic review across computational modelling approaches. Neurosci Biobehav Rev. (2023) 147:105087. doi: 10.1016/j.neubiorev.2023.105087 36791933

[27] Hauke DJ Wobmann M Andreou C Mackintosh AJ de Bock R Karvelis P. Altered perception of environmental volatility during social learning in emerging psychosis. Comput Psychiatry. (2024) 8:1. doi: 10.5334/cpsy.95 PMC11104374 38774429

[28] Kafadar E Mittal VA Strauss GP Chapman HC Ellman LM Bansal S. Modeling perception and behavior in individuals at clinical high risk for psychosis: support for the predictive processing framework. Schizophr Res. (2020) 226:167–75. doi: 10.1016/j.schres.2020.04.017 PMC7774587 32593735

[29] Cole DM Diaconescu AO Pfeiffer UJ Brodersen KH Mathys CD Julkowski D. Atypical processing of uncertainty in individuals at risk for psychosis. NeuroImage Clin. (2020) 26:102239. doi: 10.1016/j.nicl.2020.102239 32182575 PMC7076146

[30] Bloomfield MAP Mouchlianitis E Morgan CJA Freeman TP Curran HV Roiser JP. Salience attribution and its relationship to cannabis-induced psychotic symptoms. Psychol Med. (2016) 46:3383–95. doi: 10.1017/S0033291716002051 PMC5122315 27628967

[31] O’Donnell BF Skosnik PD Hetrick WP Fridberg DJ. Decision making and impulsivity in young adult cannabis users. Front Psychol. (2021) 12:679904. doi: 10.3389/fpsyg.2021.679904 34276500 PMC8280309

[32] Raine A Benishay D. The SPQ-B: A brief screening instrument for schizotypal personality disorder. J Pers Disord. (1995) 9:346–55. doi: 10.1521/pedi.1995.9.4.346

[33] Peters E Joseph S Day S Garety P. Measuring delusional ideation: the 21-item Peters et al. Delusions Inventory (PDI) Schizophr Bull. (2004) 30:1005–22. doi: 10.1093/oxfordjournals.schbul.a007116 15954204

[34] Berniker M Voss M Kording K. Learning priors for Bayesian computations in the nervous system. PloS One. (2010) 5:e12686. doi: 10.1371/journal.pone.0012686 20844766 PMC2937037

[35] Nassar MR Waltz JA Albrecht MA Gold JM Frank MJ. All or nothing belief updating in patients with schizophrenia reduces precision and flexibility of beliefs. Brain. (2021) 144:1013–29. doi: 10.1093/brain/awaa453 PMC8041039 33434284

[36] Akaike H. Information theory as an extension of the maximum likelihood principle. In: Petrov BN Csaki F Csaki BNPBF, editors. Second International Symposium on Information Theory. Academiai Kiado, Budapest (1973).

[37] Schermelleh-Engel K Moosbrugger H Müller H. Evaluating the fit of structural equation models: Tests of significance and descriptive goodness-of-fit measures. Methods psychol Res online. (2003) 8:23–74.

[38] Elston DA. Estimation of denominator degrees of freedom of F-distributions for assessing Wald statistics for fixed-effect factors in unbalanced mixed models. Biometrics. (1998), 54(3):1085–96. doi: 10.2307/2533859

[39] Dzafic I Larsen KM Darke H Pertile H Carter O Sundram S. Stronger top-down and weaker bottom-up frontotemporal connections during sensory learning are associated with severity of psychotic phenomena. Schizophr Bull. (2021) 47:1039–47. doi: 10.1093/schbul/sbaa188 PMC8266649 33404057

[40] Kreis I Zhang L Moritz S Pfuhl G. Spared performance but increased uncertainty in schizophrenia: Evidence from a probabilistic decision-making task. Schizophr Res. (2022) 243:414–23. doi: 10.1016/j.schres.2021.06.038 34272122

[41] Kreis I Zhang L Mittner M Syla L Lamm C Pfuhl G. Aberrant uncertainty processing is linked to psychotic-like experiences, autistic traits, and is reflected in pupil dilation during probabilistic learning. Cognit Affect Behav Neurosci. (2023) 23:1–15. doi: 10.3758/s13415-023-01088-2 36977966 PMC10390366

[42] Alameda-Bailén JR Salguero-Alcaniz P Merchán-Clavellino A Paíno-Quesada S. Age of onset of cannabis use and decision making under uncertainty. PeerJ. (2018) 6:e5201. doi: 10.7717/peerj.5201 30002988 PMC6034599

[43] Fridberg DJ Queller S Ahn WY Kim W Bishara AJ Busemeyer JR. Cognitive mechanisms underlying risky decision-making in chronic cannabis users. J Math Psychol. (2010) 54:28–38. doi: 10.1016/j.jmp.2009.10.002 20419064 PMC2858342

[44] Brown EC Hack SM Gold JM Carpenter WT Jr. Fischer BA Prentice KP. Integrating frequency and magnitude information in decision-making in schizophrenia: An account of patient performance on the Iowa Gambling Task. J Psychiatr Res. (2015) 66:16–23. doi: 10.1016/j.jpsychires.2015.04.007 25959618 PMC4458199

[45] O’Tuathaigh CMP Dawes C Bickerdike A Duggan E O’Neill C Waddington JL. Does cannabis use predict psychometric schizotypy via aberrant salience? Schizophr Res. (2020) 220:194–200. 32273148 10.1016/j.schres.2020.03.021

[46] Ballerini A Tortorelli M Marino P Appignanesi C Baschirotto C Mallardo L. Aberrant salience relationship with first rank symptoms. Ann Gen Psychiatry. (2022) 21:8. doi: 10.1186/s12991-022-00383-5 35172844 PMC8851729

[47] Roiser JP Howes OD Chaddock CA Joyce EM McGuire P. Neural and behavioral correlates of aberrant salience in individuals at risk for psychosis. Schizophr Bull. (2013) 39:1328–36. doi: 10.1093/schbul/sbs147 PMC3796080 23236077

[48] Howes OD Hird EJ Adams RA Corlett PR McGuire P. Aberrant salience, information processing, and dopaminergic signaling in people at clinical high risk for psychosis. Biol Psychiatry. (2020) 88:304–14. doi: 10.1016/j.biopsych.2020.03.012 32430200

[49] Jockers-Scherübl MC Wolf T Radzei N Schlattmann P Rentzsch J Gómez-Carrillo de Castro A. Cannabis induces different cognitive changes in schizophrenic patients and in healthy controls. Prog Neuropsychopharmacol Biol Psychiatry. (2007) 31:1054–63. doi: 10.1016/j.pnpbp.2007.03.006 17482741

[50] Hernaus D Xu Z Brown EC Ruiz R Frank MJ Gold JM. Motivational deficits in schizophrenia relate to abnormalities in cortical learning rate signals. Cognit Affect Behav Neurosci. (2018) 18:1338–51. doi: 10.3758/s13415-018-0643-z PMC8970346 30276616

[51] D’Souza DC Ganesh S Cortes-Briones J Campbell MH Emmanuel MK. Characterizing psychosis-relevant phenomena and cognitive function in a unique population with isolated, chronic and very heavy cannabis exposure. Psychol Med. (2020) 50:2452–9. doi: 10.1017/S0033291719002721 31615592

[52] Stefanis NC Dragovic M Power BD Jablensky A Castle D Morgan VA. Age at initiation of cannabis use predicts age at onset of psychosis: the 7-to 8-year trend. Schizophr Bull. (2013) 39:251–4. doi: 10.1093/schbul/sbs188 PMC3576149 23314189

[53] Chesney E Lawn W McGuire P. Assessing cannabis use in people with psychosis. Cannabis Cannabinoid Res. (2023) 9:49–58. doi: 10.1089/can.2023.0032 37971872 PMC10874830

[54] Freeman TP Lorenzetti V. ‘Standard THC units’: a proposal to standardize dose across all cannabis products and methods of administration. Addiction. (2020) 115:1207–16. doi: 10.1111/add.14842 31606008

[55] Wei Z Han L Zhong X Liu Y Zha R Wang Y. Chronic nicotine exposure impairs uncertainty modulation on reinforcement learning in anterior cingulate cortex and serotonin system. Neuroimage. (2018) 169:323–33. doi: 10.1016/j.neuroimage.2017.11.048 29221752

[56] Hindocha C Brose LS Walsh H Cheeseman H. Cannabis use and co-use in tobacco smokers and non-smokers: prevalence and associations with mental health in a cross-sectional, nationally representative sample of adults in Great Britain, 2020. Addiction. (2021) 116:2209–19. doi: 10.1111/add.15381 33345423

[57] Hines LA Freeman TP Gage SH Zammit S Hickman M Cannon M. Association of high-potency cannabis use with mental health and substance use in adolescence. JAMA Psychiatry. (2020) 77:1044–51. doi: 10.1001/jamapsychiatry.2020.1035 PMC7254445 32459328

